# School Burnout, Relational, and Organizational Factors

**DOI:** 10.3389/fpsyg.2019.01695

**Published:** 2019-08-02

**Authors:** Santa Parrello, Alice Ambrosetti, Ilaria Iorio, Luciana Castelli

**Affiliations:** ^1^Section of Psychology and Educational Sciences, Department of Humanities, University of Naples Federico II, Naples, Italy; ^2^Centre for Innovation and Research on Education Systems (CIRSE), Department of Education and Learning, University of Applied Sciences and Arts of Southern Switzerland (SUPSI), Locarno, Switzerland

**Keywords:** factors of school burnout, organizational identification, optimism, teachers’ well-being, comparative study

## Abstract

Work stress and burnout affect teachers to a significant extent. The objective of this study is to evaluate and compare the impact of relational and organizational factors on teacher burnout in two samples of primary school teachers, one Italian (Naples) and the other Swiss (Cantone Ticino). The hypothesis is that, given the socio-cultural and economic differences of the two contexts, the variables under investigation impact teacher burnout differently. We collected data through a self-reported questionnaire containing the following scales: Copenhagen Burnout Inventory, Satisfaction with Life Scale, Life Orientation test, organizational identification, colleague support, and workload. The Swiss sample consists of 964 teachers (26% kindergarten and 73.7% primary school teachers); the Italian sample consists of 104 teachers (20% kindergarten and 80% primary schools teachers). Descriptive analyses, mean comparison (*t* test), correlational analyses, and multiple linear regression analyses were conducted. There are no significant differences between the two samples with respect to burnout, colleague support, and workload. Correlations between burnout and the variables under investigation are significant in both samples, except for optimism in the Italian sample. Regression analysis shows that optimism and colleague support have an impact on burnout only in the Swiss sample; organizational identification has a stronger impact on burnout in the Italian sample.

## Introduction

Teachers’ well-being is a resource for schools that is constantly at risk and deserves to be protected (Hakanen et al., 2006). Recent studies on risk and protective factors of teacher burnout explore some relational and contextual aspects, such as the complexity of the educational role, the burden of responsibility, the support of colleagues, the relationship with the organization, and the social devaluation of the profession (Fiorilli et al., 2015; De Rosa et al., 2017).

In particular, the support received from colleagues (Brouwers et al., 2011) and organizational identification (Ashforth and Mael, 1989) is considered as protective factors from teacher burnout. In fact, it was found that a strong sense of organizational identification is negatively correlated to stress and burnout (Bizumic et al., 2009) and positively related to well-being (Van Dick and Wagner, 2002). Moreover, work burnout interrelates with social support (Avanzi et al., 2018) and collective efficacy (Avanzi et al., 2015).

This study analyzes and compares the impact of some relational and organizational factors on teacher burnout in two different countries, Switzerland (Ticino, the Swiss Italian-speaking Canton) and Italy. These countries differ across various socio-cultural and economic aspects, making a comparison legitimate. Indeed, as explained above, burnout does not only depend on individual factors but also on contextual factors. The following indicators partially show the contextual differences in which the teachers of the two countries carry out their daily professional activity.

First, in Ticino, kindergarten and primary schools are under the communal and the cantonal authority; in order to obtain the teaching degree, students have to obtain a bachelor’s degree in a professional university. Teaching is a highly secure job, and working conditions are good. The weekly timetable is between 32 and 25 h (depending if school meals are included); moreover, meetings and interviews with the parents have to be taken into account. In Italy, schools are under the national authority, namely the Ministry of Education. In the past, high school degree and teaching degree were mandatory in order to become a teacher; now, a 4-year bachelor’s degree is mandatory. Teaching is a highly secure job, but working conditions are quite unfavorable. The weekly timetable is of 22 h of classroom teaching, 2 h of school planning, 40 h per year for meetings and interviews with the parents, and 40 h per year for training.

With regard to working life, from the point of view of the economic situation and working conditions, Switzerland is above the OECD average with respect to the average level of disposable income per capita per year, while Italy is below the OECD average (OECD average: 30,563 USD; Switzerland: 36,378 USD; Italy: 26,063 USD). With respect to contractual wages, Italian salaries are lower than those of Switzerland and of the OECD average (OECD, 2017); the unemployment rate (IMF, 2018) in Italy is 11.26%, while in Ticino, it is 3.19% (DFE, 2019). The average life satisfaction in Switzerland is 7.9, compared to the OECD average of 7.3 and to the Italian average of 7.0 (OECD, 2017). In both countries, students (age 11–15) do not have a great deal of sympathy for the school, but Swiss students have a greater perception of the effectiveness of their schools and of the social support received in it compared to their Italian peers (HBSC, 2016). The rate of early school leaving in Italy is very high, with significant regional differences (Cederna, 2016; Parrello et al., 2019).

The aim of this study is to analyze the impact of relational and organizational factors on teacher work burnout, student-related burnout, and life satisfaction in Naples and Cantone Ticino.

The main hypothesis is that contextual factors have an impact on teacher burnout and on the relational and organizational factors related to it.

We expect that Italian teachers to have higher levels of work burnout (H1), higher levels of student-related burnout (H2), and lower life satisfaction (H3) compared to Swiss teachers. Furthermore, we assume that optimism, workload, colleague support, and organizational identification have an impact on work burnout, student-related burnout, and life satisfaction (H4).

## Materials and Methods

We examined our hypothesis on a sample that comprised two sub-samples of kindergarten and primary school teachers; the first one was composed of teachers from Naples, and the second one of teachers from Ticino.

The Italian sample was a convenience sample selected from a school located in the urban area of Naples. In Ticino, the whole population of kindergarten and primary school teachers was invited to participate in the survey (51.8% response rate).

The Swiss sample comprises 964 teachers: 710 (73.7%) of primary school, 251 (26%) of pre-primary school, and three subjects did not provide this information. In the Italian sample, 20% of teachers (*N* = 21) work in kindergarten, and the rest in primary school. In Ticino, 20% (*N* = 195) of teachers are male, while in the Italian sample, just two teachers are male.

Mean age is 42.79 (SD = 11.35) in the Swiss sample and 53.89 (SD = 7.1) in the Italian sample.

Data have been collected through a self-reported questionnaire; in Switzerland, the questionnaire was administered online; in Italy, the questionnaire was paper-pencil administered.

The following measures have been administered to both subsamples.

- The Copenhagen Burnout Inventory (CBI; Kristensen et al., 2005) has been used for measuring teacher burnout. The measure comprises three sub-scales: the personal, work, and student-related burnout scales. As suggested by Avanzi et al. (2013), we decided to use only two of three subscales – the work and the student-related burnout – being the work and the personal burnout sub-scales highly correlated and not enough discriminating.
- The work burnout scale is composed of seven items (for example: “Do you feel burnt out because of your work?”); the student-related burnout scale is composed of six items (for example: “Do you find it hard to work with students?”). Items are rated on a 5-point Likert scale, from 1 to 5, meaning 1 either “Never/almost never” or “To a low degree” and 5 either “Always” or “To a very high degree.”
- Life satisfaction was measured through the Satisfaction with Life Scale (SWLS; Diener et al., 1985; Di Fabio and Palazzeschi, 2012). The scale is composed of five items (for example: “The conditions of my life are excellent”), and responses are provided on a 7-point Likert-scale, from 1 “Strongly disagree” to 7 “Strongly agree.”
- Optimism was measured using the Life Orientation test-revised (LOT-R; Scheier et al., 1994; Giannini et al., 2008), which is a 10-item scale, composed of three items measuring optimism (for example: “In uncertain times, I usually expect the best.”), three items measuring pessimism (for example: “If something can go wrong for me, it will.”), and four items used as fillers. Responses are given on a 5-point Likert-scale, ranging from 1 “I disagree a lot” to 5 “I agree a lot.”
- Organizational identification was measured with a 6-item scale of Mael and Ashforth (1992) (Italian version: Bergami and Bagozzi, 2000) (for example: “This school’s successes are my successes”). Items are rated on a 5-point Likert scale, ranging from 1 “Totally disagree” to 5 “Totally agree.”
- In order to measure workload, we used a 6-item scale validated in Italian version by Toderi et al. (2013) based on the 8-item scale of Edwards et al. (2008) (for example: “I have unrealistic time pressures”). Responses are given on a 5-point Likert scale, ranging from 1 “Never” to 5 “Always.”
- Colleague support was measured through the scale from Edwards et al. (2008), composed of four items (for example: “I get the help and support I need from colleagues”). Responses are rated on a 5-point Likert scale, ranging from 1 “Never”, to 5 “Always.”
- Finally, we added age, gender, and a variable for investigating the familiar condition (having children or not) as control variables.

On the data collected, we first performed a comparison of means (Student’s *t* test) between the two sub-samples for burnout, life satisfaction, workload, optimism, organizational identification, and colleague support. The goal was to establish if there were statistical differences between the two samples across the variables of interest.

After a correlation analysis between the above-mentioned variables (the Pearson correlation), we performed a hierarchical linear regression in three different models on both samples.

In the first step, we added control variables, in the second step optimism and in the third step colleague support, organizational identification and workload, in order to appreciate their impact on work burnout, student-related burnout, and life satisfaction.

The regression models include the same set of variables for both samples, in order to compare the regression coefficients between the two samples.

## Results

Mean comparison shows that the two burnout measures do not differ between the two samples: teachers from Ticino and teachers from Naples show the same level of work (respectively: *M* = 2.44, SD = 0.73; *M* = 2.45, SD = 0.83, *p* > 0.05) and student-related burnout (*M* = 2.27, SD = 0.70; *M* = 2.17, SD = 0.87, *p* > 0.05).

Swiss teachers show higher levels of life satisfaction than teachers from Naples (*M* = 5.25, SD = 1.00; *M* = 4.64, SD = 1.09, *p* < 0.05). On the contrary, teachers from Naples are on average more optimistic than teachers from Ticino (*M* = 3.62, SD = 0.46; *M* = 2.63, SD = 0.45, *p* < 0.05). Moreover, teachers from Naples show higher levels of organizational identification than teachers from Ticino (*M* = 4.01, SD = 0.97; *M* = 3.70, SD = 0.75, *p* < 0.05). Regarding workload and colleague support, we could not find any statistically significant difference.

In the second step of the analysis, we estimated the Pearson correlation coefficients between all the scales, for each teacher sample (see Table 1). Correlations between teacher burnout scales and the other scales are statistically significant in both samples, except for optimism in the Italian sample. Correlations between optimism and work (*r* = −0.48, *p* < 0.05) and student-related burnout (*r* = −0.39, *p* < 0.05) are negative and significant only in the Swiss sample. Correlation coefficients between the two burnout scales and the other scales are similar between the two samples, with the exception of the organizational identification scale. In fact, correlation coefficients are *r* = −0.45, *p* < 0.05 for work burnout and *r* = −0.34, *p* < 0.05 for student-related burnout in the Italian sample, and *r* = −0.07, *p* < 0.05 and *r* = −0.12, *p* < 0.05 in the Swiss sample.

**Table 1:** Correlations between scales.

Correlations								
		Work burnout	Student-related burnout	Satisfaction with life	Optimism (life orientation)	Workload	Colleague support	Organizational identification
Ticino	Work burnout							
	Student-related burnout	0.741^**^						
	Satisfaciton with life	−0.486^**^	−0.401^**^					
	Optimism (life orientation)	−0.485^**^	−0.389^**^	0.520^**^				
	Workload	0.584^**^	0.434^**^	−0.310^**^	−0.303^**^			
	Colleague support	−0.253^**^	−0.206^**^	0.235^**^	0.240^**^	−0.209^**^		
	Organizational identification	−0.068^*^	−0.107^**^	0.109^**^	0.081^*^	−0.012	0.247^**^	
Napoli	Work burnout							
	Student-related burnout	0.778^**^						
	Satisfaciton with life	−0.421^**^	−0.450^**^					
	Optimism (life orientation)	−0.135	−0.198	0.472^**^				
	Workload	0.628^**^	0.534^**^	−0.416^**^	−0.214^*^			
	Colleague support	−0.346^**^	−0.274^**^	0.357^**^	0.293^**^	−0.119		
	Organizational identification	−0.450^**^	−0.338^**^	0.195	0.046	−0.120	0.325^**^	

* *Correlation is significant at the 0.05 level (two tailed)*.

** *Correlation is significant at the 0.01 level (two tailed)*.

The Satisfaction with Life Scale correlates significantly with all the other scales in both samples, with the exception of organizational identification in the Italian sample. Moreover, the correlation between satisfaction with life and colleague support is higher for Italian teachers than for Swiss teachers (respectively, *r* = 0.36, *p* < 0.05 and *r* = 0.23, *p* < 0.05).

Regarding the correlation between the other scales, results are similar between the two samples, with some exceptions. First, workload has a negative and significant correlation with colleague support only in the Ticino sample (*r* = −0.21, *p* < 0.05), while there is no significant correlation between the two scales in the Italian sample. Second, organizational identification is significantly correlated with satisfaction with life (*r* = −0.11, *p* < 0.05) and optimism (*r* = 0.08, *p* < 0.05) only in the Swiss sample, while there are not statistically significant correlations in the Italian sample.

All the other scales are positively correlated with each other, with the exception of workload and organizational identification that do not show significant correlations in both samples (see Table 1).

In order to better establish the relationship between burnout, life satisfaction, and the other variables under investigation, we performed a hierarchical linear regression in three steps (Table 2).

**Table 2:** Regressions results.

	Work burnout – Model 1		Student-related burnout – Model 2		Satisfaction with life – Model 3	
	Ticino	Naples	Ticino	Naples	Ticino	Naples
**Step 1**						
Gender	−0.02	−0.19	−0.18^***^	0.14	0.08^***^	0.12
Age	0.00	0.02^**^	0.00	0.03^**^	−0.01^***^	0.09
Dependent children	−0.08^*^	0.00	0.01	−0.14	0.22^***^	−0.02
**Step 2**						
Optimism	−0.51^***^	0.12	−0.43^***^	0.01	0.99^***^	0.83^***^
**Step 3**						
Workload	0.51^***^	0.67^***^	0.35^***^	0.59^***^	−0.21^***^	−0.42^***^
Colleague support	−0.06^***^	−0.17	−0.04	−0.14	0.09^***^	0.25
Organizational identification	−0.02	−0.27^***^	−0.06*	−0.17	0.06	−0.01
Intercept	2.786^***^	0.802	2.873^***^	0.322	2.805^***^	2.640
*R*^2^ Step 1	0.01^***^	0.07	0.02^***^	0.09	0.03^***^	0.07
Δ*R*^2^ Step 2	0.23^***^	0.01	0.15^***^	0.02	0.26^***^	0.2^***^
Δ*R*^2^ Step 3	0.21^***^	0.46^***^	0.11^***^	0.29	0.03^***^	0.1^***^
*R*^2^ total	0.45^***^	0.54^***^	0.29^***^	0.39^***^	0.32^***^	0.37^***^

*The β coefficients reported are relative to Step 3. Independent variables: gender 0 = male, 1 = female; dependent children 0 = no, 1 = yes*.

* p < 0.05;

** p < 0.01;

*** *p < 0.001*.

The first model shows that in the Swiss sample, age and gender do not have any statistically significant impact. Instead, having children seems to have a modest but positive impact on work burnout: Swiss teachers with children show a lower level of work burnout than the ones having one child or more (*β* = −0.08, *p* < 0.05). The same result is not found in the Italian sample. Age is a predictor of work burnout for Italian teachers (*β* = 0.02, *p* < 0.05).

A difference is also found for optimism: the effect is quite strong for Ticino’s teachers (*β* = −0.51, *p* < 0.05), whereas for the Italian sample, this variable has no effect on the work burnout. Likewise, for colleague support, we did not find an effect for the sample from Naples, but a statistically significant one in the Ticino sample. Namely a high perceived colleague support corresponds, on average, to a high work burnout (*β* = −0.06, *p* < 0.05). At the opposite, organizational identification seems to play a role in predicting work burnout only in the Naples sample (*β* = −0.27, *p* < 0.05). Finally, workload has an effect in both samples: teachers with high values of workload have, on average, high values of work burnout (*β* = 0.51, *p* < 0.05 for Ticino, *β* = 0.67, *p* < 0.05 for Naples).

The first model explains, in total, 45% of the variance for the Swiss sample and 54% for the Italian sample. Interestingly, for Naples, adding the third block substantially increases the predictive power (Δ*R*^2^ = 0.46), as if in this sample, the control variables and optimism barely explain work burnout. For Ticino, the second and the third blocks seem to explain the same share of variance (respectively, Δ*R*^2^ = 0.23 and Δ*R*^2^ = 0.21).

From the second model, which has student-related burnout as the dependent variable, different results with respect to the first model emerge. In fact, gender is statistically significant for Ticino, whereas age and having children are no longer statistically significant. Women are thus more likely, all other things being equal, to have a lower level of student-related burnout than male teachers (*β* = −0.18, *p* < 0.05). For the Italian sample, the control variables have the same impact as in the first model, particularly with age having a negative impact (*β* = 0.03, *p* < 0.05).

For optimism, we also found the same results as in Model 1, with these variables having an effect on student-related burnout only in the Swiss sample (*β* = −0.43, *p* < 0.05). That is, teachers with high values of optimisms are less likely to have high values of student-related burnout. Regarding the other three scales introduced, we found again an effect of workload in both samples (*β* = 0.35, *p* < 0.05 for Ticino, *β* = 0.59, *p* < 0.05 for Naples) and organizational identification is now significant for Ticino (*β* = −0.06, *p* < 0.05), whereas it is no more significant for Naples. Colleague support is not significant in both samples.

This model explains 29% of the variance for the Swiss sample and 39% for the Italian sample. Values are lower than in the first model, meaning that the set of variables introduced in the models better explains the work burnout dependent variable. Again, for the Italian sample, the salient variables are the ones included in the third block (Δ*R*^2^ = 0.29). As in the first model, the second and the third blocks of variables in the Swiss sample seem to contribute in the same proportion (Δ*R*^2^ = 0.15 and Δ*R*^2^ = 0.11).

Finally, the third model has life satisfaction as dependent variable. For the Italian sample, we found no significant effect of the control variables, while for the Swiss sample, age and having children have an impact on the satisfaction with life. In fact, older teachers seem to have lower values of satisfaction with life (*β* = −0.01, *p* < 0.05), and having children increases the satisfaction with life (*β* = 0.22, *p* < 0.05), other things held constant. Optimism has a strong and positive effect in both samples: teachers with high values of optimism have in mean a high satisfaction with life (*β* = 0.99, *p* < 0.05 for Ticino; *β* = 0.83, *p* < 0.05 for Naples).

Workload has also an impact on life satisfaction in the two samples: the effect seems stronger for the Italian teachers (*β* = −0.42, *p* < 0.05 vs. *β* = −0.21, *p* < 0.05). Colleague support has an impact only on the Swiss sample (*β* = 0.09, *p* < 0.05), whereas in the Italian sample, there is no effect of this on life satisfaction. Finally, organizational identification has no impact on both the samples.

The model explains 32% of the variance for the Swiss sample and 37% for the Italian sample. As in the other two models, the third block is the one that explained mainly satisfaction with life (Δ*R*^2^ = 0.37) in the Italian sample. On the contrary, for Ticino, the variable that better explains life satisfaction is optimism (Δ*R*^2^ = 0.26).

## Discussion

Results confirm what has already been found in the literature, namely the impact of contextual and organizational factors on the teachers’ well-being and burnout (Castelli et al., 2017).

In contrast to what has been assumed, i.e., higher levels of burnout are expected in the Italian sample than the Swiss sample, burnout, perceived support, and perceived workload are present to a similar extent in the two samples, despite the existing differences in terms of the socio-economic context, wages and working conditions. Therefore, it seems that the macro factors of the work context have a limited impact on the risk of developing burnout at work.

At the same time, results have highlighted some interesting differences between the populations under study, which could also be attributable to the differences already highlighted regarding the working conditions of the two samples: Italian teachers show a higher level of optimism and identification with their school than their Swiss colleagues who are instead more satisfied with their lives.

With respect to the correlations between the variables identified (dispositional, relational, and organizational), they are all related to burnout in both contexts, confirming the initial assumptions.

Compared to what emerges from the regression analysis, where the impact of the variables under investigation on work burnout was studied, it is very interesting to find some differences between the two samples: optimism has a significant effect on teachers in Switzerland, while for the Italian sample, this variable has no effect on work burnout. Similarly, for the support of colleagues, we did not find an effect in the Italian sample, but it is statistically significant in the Swiss sample. This would lead us to think of a difference in professional culture between the two samples considered, where Swiss teachers seem to rely more on social support than Italian teachers.

On the contrary, organizational identification seems to play a role in predicting burnout only in the sample of Naples.

Finally, the workload has a significant effect in both samples examined, confirming previously results from other studies (Castelli et al., 2017): teachers with a high workload have, on average, high values of work burnout.

If the workload therefore has an effect on both samples, another difference can be seen in the effect of optimism and organizational identification In fact, optimism has a significant effect only in the Swiss sample on the three dependent variables, whereas in the Italian sample, optimism has an effect only in the life satisfaction. Thus, it seems that in the Swiss sample, an individual disposition protects teachers from burnout, while for the Italian sample, an organizational variable seems to be more protective. It is interesting to assume, thus, that it is precisely the dimension of organizational identification that compensates for a greater presence of risk factors for Italian teachers, in line with what has already been highlighted in other works previously mentioned (Avanzi et al., 2018).

In conclusion, this study allowed to explore the protective factors of work burnout and student-related burnout in two samples that significantly differ for their working contexts and environments. However, it is important to underline some limitations of the study. The first limitation concerns the sampling procedure: the Italian sample was a convenience sample; thus, the results obtained may not be generalized to the whole population of Italian teachers. As stated before (Wilkinson and Task Force on Statistical Inference APA Board of Scientific Affairs, 1999), the use of convenience sample could be considered as acceptable, but results must be read carefully.

The second limitation regards the interpretation of the results: a causal relation between the variables under investigation has been postulated, while, in fact, the regression model does not exclude the possibility of a reverse causality; also, for this reason, results have to be read carefully.

Finally, it is possible that we omitted variables that could explain our dependent variables and that are also strongly correlated to our independents variables.

The limitations listed above suggest that a more robust sampling procedure and considering further variables in the analysis would represent potential directions for future investigations. Moreover, it would be interesting to explore the existence of mediation effects between the variables under investigation.

## Data Availability

The data sets generated for this study are available upon request from the corresponding author.

## Ethics Statement

The studies that involved human participants were reviewed and approved by the Ethics Committee for the Psychological Research (CERP) of the Department of Humanities at the University Federico II of Naples. Patients/participants have provided their written informed consent to participate in this study.

## Author Contributions

All authors designed the study. AA and II analyzed the data. All authors interpreted the results, wrote up the first draft of the manuscript, and approved the final version.

### Conflict of Interest Statement

The authors declare that the research was conducted in the absence of any commercial or financial relationships that could be construed as a potential conflict of interest.
